# Nulliparity enhances the risk of second primary malignancy of the breast in a cohort of women treated for thyroid cancer

**DOI:** 10.1186/1477-7819-9-88

**Published:** 2011-08-12

**Authors:** Fabrizio Consorti, Gianluca Di Tanna, Francesca Milazzo, Alfredo Antonaci

**Affiliations:** 1 Dept. Of Cardiocirculatory Pathophysiology, Anesthesiology and General Surgery - "Sapienza" University of Rome, Italy; 2 Dept. Of Experimental Medicine - "Sapienza" University of Rome, Italy

**Keywords:** thyroid cancer, breast cancer, second primary malignancy, risk factor

## Abstract

**Background:**

Many studies have reported an increased risk of developing a second primary malignancy (SPM) of the breast in women treated for thyroid cancer. In this study, we investigated several potential risk factors for this association. The aim of this retrospective cohort study was to identify a subgroup of women surgically treated for papillary thyroid cancer that may benefit from more careful breast cancer screening.

**Methods:**

A total of 101 women surgically treated for papillary thyroid cancer from 1996 to 2009 with subsequent follow-up were interviewed by phone regarding personal risk factors and lifestyle habits. Only 75 questionnaires could be evaluated due to a 25.7% rate of patients not retrieved or refusing the interview. Data analysis was performed using a multivariate logistic model.

**Results:**

The standardised incidence ratio (SIR) for breast cancer was 3.58 (95% IC 1.14 - 8.37). Our data suggest a protective effect of multiparity on the development of a SPM of the breast (O.R. 0.15; 95% IC 0.25 - 0.86). Significant associations were not found with other known risk factors including Body Mass Index (BMI), age at first tumour, concurrent metabolic diseases, smoking, physical activity and familiarity.

**Conclusions:**

This study confirms that a higher incidence of SPM of the breast is observed in women treated for papillary thyroid cancer. Additionally, this risk is increased by nulliparity, thus a strict breast screening program for nulliparous women treated for thyroid cancer may be advisable.

## Background

Papillary thyroid cancer is the most commonly observed endocrine neoplasm. Its overall age standardised rate in Italy is 9.1 cases/100.000 persons/year, even if its incidence is higher in women (14.3). The mortality rate is much lower, accounting 0.4 death/100.000 [1]. Because thyroid cancer survivors may live for several decades following diagnosis, they may develop a second primary malignancy (SPM). In a meta-analysis conducted by Subramanian *et al.*, 1409 publications were reviewed, and they found that breast carcinoma is the most frequent SPM in women treated for thyroid carcinoma [2]. Several large studies analysed the incidence of SPM of the breast using cancer registries [3-5], but these studies did not focus on possible risk co-factors, such as the lifestyle or family and past medical history of the women. The present study examined common risk factors for breast carcinoma in a cohort of women surgically treated for papillary thyroid carcinoma. Based on these factors, our goal was to identify a subgroup of women with an increased risk of developing a SPM of the breast that may benefit from more careful mammary screening.

## Methods

Women with a histological diagnosis of papillary thyroid cancer and available follow-up data were selected from a database of patients surgically treated from 1996 to 2009 for thyroid disease at one of the Departments of Surgery of Policlinico Umberto I in Rome. A total of 101 women with papillary thyroid cancer were present in the database and were all included in this retrospective cohort study. A questionnaire was designed to guide a phone interview to investigate patients' personal risk factors and lifestyle habits (additional file 1). All patients were called and interviewed by the same individual. Each interview lasted approximately ten minutes. In total, 75 interviews were conducted. Of the selected 101 women, 26 of these patients (25.7%) were unavailable for interviews (2 did not survive, 22 could not be reached via phone and 2 refused the interview).

Data analysis was performed using univariate and multivariate logistic models. In addition, the ratio between expected and observed incidence of breast cancer during the follow-up period was calculated (SIR: standardised incidence ratio = expected/observed). Confidence intervals were calculated from a poissonian distribution for standardised rates, according to [6].

## Results

Of the 75 patients who answered the questionnaire, the mean age at the diagnosis of papillary cancer was 57.35 ± 12.75 years, with a mean follow-up period at the moment of the interview of 74.52 ± 12.75 months (6.21 ± 1.06 years). In the follow up period after the diagnosis of papillary thyroid cancer, 7 SPMs of the breast were observed. All these 7 patients had been already operated at the moment of the interview and their diagnosis of breast cancer had been histologically confirmed. The mean time interval between the diagnosis of thyroid cancer and the subsequent diagnosis of breast cancer was 5.80 ± 3.24 years. Two cases of detected breast cancer occurred within 2 years from the papillary carcinoma diagnosis and they were not considered in the SIR calculation. According to many other similar studies[3,4], these cases were considered to occur concurrently to thyroid cancer.

For SIR calculation we considered an expected incidence of 300 new cases/y/100000 women, (i.e., 0.225 new cases/y/75 women). This incidence is based on Piscitelli *et al. *[7], which was chosen because this study gave us a good estimate of the incidence in an Italian population, in the same time period as our study and for a population similar in age. During the follow-up period, the expected incidence was 1.40 cases (0.225*6.21); however, we actually observed 5 cancer cases, resulting in a calculated SIR of 3.58 (95% IC 1.14 - 8.37). Univariate analysis indicated a protective effect of multiparity on the onset of a breast SPM (*O.R*. 0.15; 95% IC 0.25 - 0.86) (Table 1). Parity was also an independent risk factor based on multivariate analysis. Significant associations were not detected with other known risk factors, such as BMI, age at first tumour, concurrent metabolic diseases, smoking, physical activity and family history (Table 1).

**Table 1:** Logistic regression analysis of measured factors

Variable	Univariate analysis		Multivariate analysis	
	**Odds ratio (95% CI)**	**P value**	**Odds ratio (95% CI)**	**P value**

**Age at first tumor**	1.06 (0.98 - 1.14)	0.14	1.07 (0.97 - 1.18)	0.16

**Weight**	0.96 (0.89-1.04)	0.33	0.98* (0.90-1.07)	0.65*

**Child**	0.15 (0.03-0.86)	0.03	0.08 (0.01-0.94)	0.04

**Smoke**	5.33 (0.59-48.33)	0.72	4.79 (0.43-53.25)	0.20

**Physical activity**	0.32 (0.03-2.88)	0.31	0.17 (0.01-4.15)	0.28

**Concurrent metabolic diseases**	0.82 (0.15 - 4.40)	0.82	0.08 (0.00 - 1.56)	0.10

**Family history of breast cancer**	2.65 (0.49-14.41)	0.26	1.06*(0.10 - 0.88)	0.96*

The multivariate model considered as the most accurate includes as selected covariate only: age at first tumor, child, smoke, physical activity and concurrent metabolic diseases, expressed as dichotomous variables (yes/no). Weight and family history were excluded due to high p-value i.e. p > 0.6.

Hosmer Lemeshow test for goodness of fit was not statistically significant (p = 0.07), indicating a good quality of the model itself.

* the odds ratios for weight and family history of breast cancer are from the "full" model which includes all the covariates in the table.

The characteristics of the study population are provided in Table 2.

**Table 2:** Distribution of risks factors, life-style habits and past medical history in the sample

Characteristic	Value
BMI (Body Mass Index) *mean ± SD*	26.83 ± 4.74

Education level *n (%)*	
Degree	11 (14.67)
High school	31 (41.33)
Primary school	33 (44.00)

Parity *n (%)*	64 (85.33)

Smoke *n (%)*	
Lifetime nonsmoker	37 (49.33)
Current smoker	10 (13.34)
Former smoker	28 (37.33)

Alcohol use *n (%)*	
Nondrinker	22 (29.33)
Drinker	53 (70.67)

Physical activity *n (%)*	
None	48 (64.00)
1-2 times/week	13 (17.33)
>2 times/week	14 (18.67)

Menopause *n (%)*	55 (73.33)

Menarche *mean age ± SD*	12.38 ± 1.33

Concurrent metabolic diseases *n (%)*	
Dyslipidemia	3 (4.00)
Diabetes type 2	1 (1.33)
Hypertension	13 (17.33)
Metabolic syndrome	9 (12.00)
Diabetes type 2 and dyslipidemia	1 (1.33)
Diabetes type 2 and hypertension	1 (1.33)
Dyslipidemia and hypertension	13 (17.33)
None	34 (45.35)

Family history of thyroid cancer *n (%)*	15 (20.00)

Family history of breast cancer *n (%)*	33 (44.00)

SPM (Second Primary Malignancy) *n (%)*	
Breast	7 (9.33)
Urinary bladder	1 (1.33)
Uterus	2 (2.67)
None	65 (86.67)

Benign breast disease *n (%)*	
Fibroadenoma	6 (8.00)
Mastopathy	20 (26.67)

Twenty-four patients (32%) did not follow a regular breast screening program. Because we only conducted a phone interview and not a clinical exam of the women, the actual number of SPMs of the breast could have been underestimated.

Additionally, 72 of the 75 patients underwent radioactive iodine therapy.

## Discussion

### Risk quantification

The incidence of thyroid cancer has almost doubled in Italy between the periods 1991-1995 and 2001-2005. This trend is almost exclusively due to an increase in papillary thyroid cancer (PTC) [8]. The mortality rate for patients diagnosed with thyroid cancer, however, has declined [9], and the 15 year survival rate is approximately 80% [10]. Due to this prolonged survival time, development of a SPM is becoming an actual possibility for thyroid cancer survivors. For example, it is known that women treated for thyroid cancer have an increased incidence of subsequent breast cancer compared to the general population [11], and our research confirms this finding. We identified 7 patients with breast cancer among the 75 women previously treated for papillary thyroid cancer. Five of the cases were documented more than two years from the initial diagnosis of PTC. The mean follow-up period was 6.21 years, resulting in a SIR of 3.58 (95% IC 1.14-8.37).

In a meta-analysis conducted by Subramanian *et al. *[2], a pooled SIR of 1.25 (95% CI 1.17-1.32) was calculated for breast SPMs from a group of 83292 patients. This SIR was lower than our calculated SIR. It is important to note that the sample size used in our study was smaller and our confidence interval was larger than those calculated in the meta-analysis, but our study includes the SIR value of the meta-analysis (i.e., 1.25). Furthermore, we found that 24 of the 75 women (32%) questioned did not follow a regular breast screening program; therefore, the number of breast SPMs detected could have been an underestimate. Additionally, Adjadj *et al. *[4] observed an increased risk of breast cancer among thyroid cancer patients and was unable to detect a significant correlation with treatment (radiotherapy or radioactive iodine therapy) of the initial cancer. Because the mammary gland has the same sodium-iodine symport as the thyroid gland, radioactive iodine could be implicated in breast carcinogenesis. Additionally, radiotherapy treatment could induce women of childbearing age to delay pregnancy, and parity is a known protective factor for breast cancer, especially in younger women [12].

Although Canchola *et al. *[4] detected a higher incidence only of *in situ *SPM breast cancer, we observed an increased risk of invasive breast cancer. Chen *et al. *(2001) [3] and Brown *et al. *(2008) [11] observed a higher incidence of SPM of the breast in 25-49-year-old women previously treated for thyroid cancer than in older women. Despite these results, we were unable to detect a significant correlation between the incidence of breast cancer and young age. It is important to note that the mean age at first tumour diagnosis in our sample population was 57.35 ± 12.75 years. This mean age was higher than that observed in the series from Chen *et al. *and Brown *et al.*, reflecting the general trend for the mean age at tumour onset in Italian populations [9].

According to Rubino *et al. *and Sawka *et al. *[13,14], thyroid cancer patients treated with radioactive iodine have an increased risk of developing several SPMs, including leukemia or bone cancer, but not breast cancer. Because 72 of the 75 patients (96%) were treated with radioactive iodine, we were unable to assess the correlation between breast SPM risk and radioactive iodine treatment in our study.

### Risk factors

Previously, the majority of studies on the incidence of SPM following thyroid cancer were based on cancer registries. Consequently, they could not take detailed personal data into account, and important information such as life style and personal risk factors of patients were not analysed. Using phone interviews, we investigated several potential risk factors involved in the association between thyroid and subsequent breast cancers. The aim of this retrospective cohort study was to identify a subgroup of women surgically treated for papillary thyroid cancer that may necessitate a more careful mammary screening.

Based on our analysis, we observed a significant protective effect of multiparity for SPM of the breast. Obesity has been associated with increased incidence of thyroid cancer [15]. In our study, the majority of patients were overweight with a mean BMI of 26.83 ± 4.74 Kg/m^2^. We were unable to detect a significant association between weight and risk using both univariate and multivariate analyses. Despite the fact that women with a first-degree relative suffering from breast cancer have a higher risk of developing primary breast cancer [12], a significant association was not detected with family history.

### Study limitations and perspectives for the future

This retrospective cohort study only included 101 patients, thus a larger patient population is needed to confirm our findings. Furthermore, we were unable to detect a significant association between young age and SPM, but the mean age for our sample population was higher than those reported in other studies [3,4,10].

Age of the first live birth as well as the number of children could be relevant information. Nevertheless, in this study we tried to keep the interview as short and simple as possible and to consider only dichotomous variables for calculation. Starting now from the consideration that childbearing is a risk factor, more addressed and large studies are needed to define in details extent and meaning of this finding. An additional limitation to the study was the loss of 25.7% of the initial 101 patients due to interview unavailability. It is possible that our results may have been different if the other 22 interviews had been collected, even if there is not a reason to presume a specific direction for this potential bias. In fact, personal characteristics of this sub-group, as recorded in the database, were similar to the rest of the sample.

The pathophysiologic relationship between thyroid and breast cancer is not clearly understood. Potential hypotheses for the link between these tissues include a genetic predisposition, dysregulation of the immune system or a hormonal cause. Additionally, the observed increased incidence of breast SPM could be artificial because women previously treated for cancer may be more cautious with their health.

Hall *et al. *[16] demonstrated *in vitro *that thyroid hormones (T3) may function to mimic or enhance the effect of oestrogens on breast cancer cell proliferation. Based on this information, it is clear that the effect of high l-thyroxine doses on breast tissue during suppressive hormonal therapy following thyroidectomy in thyroid cancer patients should to be investigated. According to the experimental hypothesis that high l-thyroxine doses could enhance the risk of breast cancer, hormone replacement therapy for postmenopausal women should also be investigated as a possible risk factor. This information was too detailed to be collected by a phone interview in this study, because of the large number of commercially available products.

## Conclusions

This study confirms that there is an increased incidence of breast SPM in women treated for papillary thyroid cancer. With the exception of nulliparity, we were unable to detect significant associations between the incidence of SPM and several known risk factors. From a methodological perspective, the questionnaire design was appropriate. It allowed for a detailed phone interview that only required 10 minutes of time from each patient. Based on our findings, a strict breast screening program for nulliparous women treated for thyroid cancer may be advisable. Although we were able to detect a significant association, larger studies are necessary to confirm our findings and investigate other potential risk factors (e.g., suppressive hormonal therapy).

## Competing interests

The authors declare that they have no competing interests.

## Authors' contributions

FC and AA operated the patients and did the follow up, FM designed the questionnaire and did the interviews, GDT was responsible for the epidemiological design and statistics. FC and FM wrote the paper, AA and GDT reviewed the text. All authors read and approved the final manuscript.

## Supplementary Material

Additional file 1 **the questionnaire for the interview**. a 25 items questionnaire to collect information about risk factors related to personal habits and life-style. Tested for a phone interview of about 10 minutes. Click here for file
