# Supplementary material for: Nulliparity enhances the risk of second primary malignancy of the breast in a cohort of women treated for thyroid cancer
Source: World J Surg Oncol. 2011 Aug 12;9:88. doi: 10.1186/1477-7819-9-88 (PMC3174118; doi:10.1186/1477-7819-9-88)
Supplement: Additional file 1 — the questionnaire for the interview. a 25 items questionnaire to collect information about risk factors related to personal habits and life-style. Tested for a phone interview of about 10 minutes. [file 1477-7819-9-88-S1.DOC]

**QUESTIONNAIRE**

Short sentence of introduction and presentation of the goal of the interview.

1. How much do you weight?
2. How tall are you?
3. What is your school qualification?
4. Which is your occupation?
5. Have you children?

- how many?

1. What about your smoke habits? never? are you still smoking?

- which was your age at the beginning of your smoking?

If you stopped smoking:

- how many years ago did you stop smoking?

- why?

1. Do you practice any physical activity?

- which kind of activity?

- ho often? (never, 1-2 times/week, > 2 times/week)

1. What about your alcohol consumption?

- what do you drink: wine, beer, liquor?

- how many glasses? (0 , ≤7/week, >7/week)

1. Do you use salt with added iodine?

If yes: occasionally or exclusively?

1. Are you vegetarian?

- since when?

1. Are you in menopause?

If yes:

- at which age did menopause occur?

1. At which age had you your first period?
2. Do you take any medication? e.g. for hypertension, for diabetes, for a high level of fat in the blood?
3. Have you had any other tumor beyond your tumor to the thyroid?

If yes:

- where? can you remember which kind of tumor?

1. Have you had any disease of the blood cells?

If yes:

- can you remember which kind of disease?

1. Did you ever do a radiation therapy when younger?
2. As far as you know, are there genetic diseases in your family?
3. As far as you know, are there cases of tumor of thyroid gland in your family?
4. As far as you know, are there cases of tumor of the breast in your family?
5. After the operation to the thyroid, did you do radiation therapy with radioactive iodine?
6. Did you do any other treatment for your tumor of the thyroid, also in other hospitals?
7. Do you do regular follow up visits after the operation to the thyroid?
8. Have you had any disease of the brest?

If yes:

- which kind of disease?

- When was the diagnosis done?

If the diagnosis was carcinoma:

- have you been operated?

- did you do chemotherapy?

- did you do radiation therapy?

- did you do hormone therapy?

- have you any problems with the other breast?

- do you do regular follow up visits after the operation to the breast?

1. Are you used to perform a mammography?

- Yearly? every two years? more than that?

1. When did you perform your last mammography?
